# Effect of isoniazid preventive therapy on tuberculosis incidence and associated risk factors among HIV infected adults in Tanzania: a retrospective cohort study

**DOI:** 10.1186/s12879-019-3696-x

**Published:** 2019-01-17

**Authors:** Amon Sabasaba, Henry Mwambi, Geoffrey Somi, Angella Ramadhani, Michael J. Mahande

**Affiliations:** 1 Department of Epidemiology and Biostatistics, Institute of Public Health, Kilimanjaro Christian Medical University College, P.O.Box 2240, Moshi, Tanzania; 2 School of Statistics and Actuarial Sciences, University of Kwazul-Natal, Durban, South Africa; 3 Ministry of Health, Community Development, Gender Elderly and Children (MOHCDGEC) - National Aids Control Program, Dar es salaam, Tanzania

**Keywords:** TB incidence, Isoniazid preventive therapy, Dar es salaam, Tanzania

## Abstract

**Background:**

Tuberculosis (TB) continues to be the leading cause of morbidity and mortality among human immunodeficiency virus (HIV) infected individuals in Sub Saharan Africa including Tanzania. Provision of isoniazid preventive therapy (IPT) is one of the public health interventions to reduce the burden of TB among HIV infected persons. However there is limited information about the influence of IPT on TB incidence in Tanzania. This study aimed at ascertaining the effect of IPT on TB incidence and to determine risk factors for TB among HIV positive adults in Dar es Salaam region.

**Methods:**

A retrospective cohort study was conducted using secondary data of HIV positive adults receiving care and treatment services in Dar es Salaam region from 2011 to 2014. TB incidence rate among HIV positive adults on IPT was compared to those who were not on IPT during the follow up period. Risk factors for incident TB were estimated using multivariate Cox proportional hazards regression model.

**Results:**

A total of 68,378 HIV positive adults were studied. The median follow up time was 3.4 (IQR = 1.9–3.8) years for patients who ever received IPT and 1.3 (IQR = 0.3–1.3) years among those who never received IPT. A total of 3124 TB cases occurred during 114,926 total person-years of follow up. The overall TB incidence rate was 2.7/100 person-years (95%CI; 2.6–2.8). Patients on IPT had 48% lower TB incidence rate compared to patients who were not on IPT (IRR = 0.52, 95%CI; 0.46–0.59). Factors associated with higher risk for incident TB included; being male (aHR = 1.8, 95% CI; 1.6–2.0), WHO stage III (aHR = 2.7, 95% CI; 2.3–3.3) and IV (aHR = 2.4, 95% CI; 1.9–3.1),being underweight (aHR = 1.7, 95% CI; 1.5–1.9) while overweight (aHR = 0.7, 95% CI; 0.6–0.8), obese (aHR = 0.5, 95% CI; 0.4–0.7), having baseline CD4 cell count between 200 and 350 cells/μl (aHR = 0.7, 95% CI; 0.6–0.8) and CD4 count above 350 cells/μl (aHR = 0.5, 95% CI; 0.4–0.6) were associated with lower risk of developing TB.

**Conclusion:**

Isoniazid preventive therapy (IPT) has shown to be effective in reducing TB incidence among HIV infected adults in Dar es Salaam. More efforts are needed to increase the provision and coverage of IPT.

## Background

Despite the availability of antiretroviral therapy (ART), Tuberculosis (TB) is the most common presenting illness among people infected with Human Immunodeficiency syndrome Virus (HIV) [1]. People living with HIV are at about thirty times higher risk of developing TB compared to non-HIV infected individuals [2]. HIV co-infection have been associated with unusual presentations of TB such as smear negative and abnormal chest radiographs thus causing a diagnostic challenge, poor treatment outcome and subsequent increased mortality [3, 4]. In 2017, the World health Organization (WHO) estimated that approximately 10 million people developed TB globally [2]. Of these 9million were adults (5.2 million were male, 3.8 million females) and 1 million were children. During the same period, about 1.3 million HIV negative patients were reported to die of TB, whereas additional 300,000 TB deaths were from HIV infected patients [2].

Isoniazid preventive therapy (IPT) together with other interventions such as intensified case finding and infection control have been widely recommended to reduce the burden of TB in people living with HIV [5]. IPT has been proven to be safe with minimal and less frequently reported side effects such as hepatotoxicity and gastrointestinal symptoms [6]. Studies have shown that IPT can lower TB incidence among PLHIV by up to 70% if used with or without ART [7, 8]. However, uptake of IPT has been relatively low in most developing countries including Tanzania [9].

In Tanzania, IPT was widely scaled up in 2011 whereby phase one of the roll out involved ten regions including Dar es salaam [10]. To date it is not well documented to what extent has IPT influenced the TB incidence in Tanzania. Furthermore, little is known on other associated risk factors for TB among HIV positive adults enrolled in care and treatment clinics in Tanzania. This study aims at ascertaining the effect of IPT by comparing TB incidence rates among patients on IPT compared to those not on IPT using routinely collected secondary data. These information are important to policy makers and clinicians to help evaluate the effectiveness of IPT and associated changes in TB incidence in Tanzania particularly Dar es salaam region which is highly endemic for both TB and HIV [11, 12].

## Methods

### Study design and setting

This is a retrospective cohort study which was conducted using de-identified secondary data of HIV positive patients enrolled in fifty (50) Care and treatment clinics (CTCs) in Dar es Salaam region between 2011 and 2014. The study was done in Dar es Salaam region which is the largest city in Tanzania with three districts/municipalities namely Temeke, Ilala and Kinondoni. Dar es Salaam region is the commercial center with an estimated population of 5 million inhabitants. The region is endemic for both TB and HIV as it contributes about 22% of all TB notified cases in Tanzania [11] and has an HIV prevalence of 4.7% which is slightly below the national average of 5.0% [12].

### Study population and period

The study comprised of all HIV positive adults (≥15 years) who were enrolled in CTCs of Dar es Salaam region for the first time between January 2011 to December 2014. All patients aged below 15 years, those with missing information on age and those patients who were diagnosed with TB or had documentation of taking Anti TB medications during enrolment were excluded.

### Data source and study variables

We used de-identified secondary data from the electronic database at National Aids Control Program (NACP) which stores routine collected HIV information for patient clinical monitoring and reporting to the government. All patients enrolling in HIV care were registered in the database. Variables were extracted from the database to form a secondary dataset which was used for this analysis. All patients enrolled during the study period were included in the study.

According to the National guideline, it is recommended that for management of HIV all HIV patients attending CTCs should be screened for TB at each clinic visit. This is done using a standard screening algorithm which starts with the use of symptomatic tool and where needed sputum for Acid Fast Bacilli (AFB) and/or a chest radiograph is taken. For this study, the primary outcome was “incident TB” documented in the dataset as a patient newly diagnosed with TB by either Acid-Fast Bacilli sputum smear or chest radiograph or prescribed anti-TB medications during follow-up. The primary exposure was history of using IPT. As per Tanzanian national guideline for management of HIV, eligibility criteria for IPT initiation includes all HIV positive adults without signs and symptoms of active TB as well as those treated for TB more than two years earlier. IPT is given at a dose of 300 mg daily for six to nine months and should be repeated after two years from the first dose of the IPT cycle. During follow up, some of the patients became eligible for IPT or ART and some were initiated while others were not for reasons we couldn’t ascertain from the data.

Other exposure variables included age, sex, weight, height, baseline CD4 count, baseline WHO clinical staging, history of cotrimoxazole use, history of ART use, and other important demographics parameters such as date of enrollment, date of each visit, date of IPT, ART and cotrimoxazole initiation.

### Sampling and sample size estimation

**Fig. 1:**
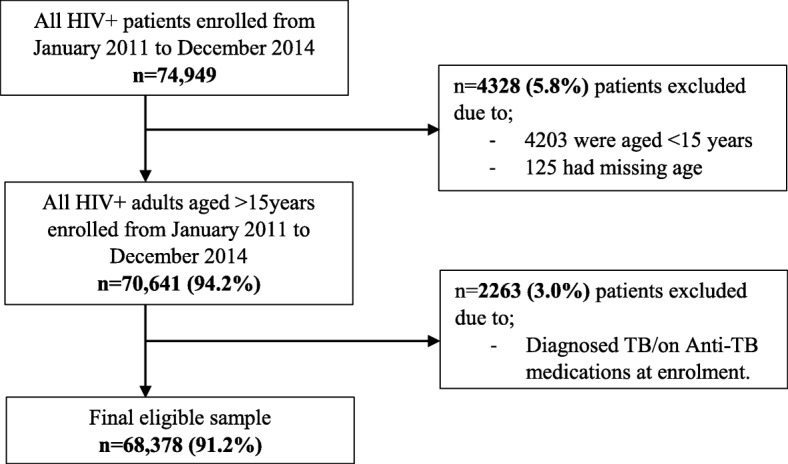
Flow chart showing the recruitment of the study population

We used records of all patients who enrolled in care and treatment clinics of Dar es Salaam region between January 2011 and December 2014 which made a total of 74,949 HIV positive patients. Of these, 5.8% (4328) records were excluded because they were aged less than 15 years and some had missing information on age. Of the remaining records, 2263 (3.2%) were excluded as they were either diagnosed for TB or on Anti TB medications at enrolment as summarized in the flow chart below. The process of obtaining the final sample is summarized in the flow diagram (Fig. 1**)** below.

### Data analysis

Data analysis was performed using Stata version 13.1 (Stata Corp, College Station, TX). Patient-time at risk for TB was defined as time from when the client was firstly enrolled in the CTC to the date of first diagnosis of TB, the last date when the patient visited clinic or 31st December 2014 (i.e censoring date) whichever occurred first. A variable for IPT use was treated as time-updated variable whereas a patient in IPT group accrued person time when initiated IPT. Descriptive statistics were summarised using means (SD) and medians (IQR) for continuous variables; frequency and proportion for categorical variables. Kruskal-Wallis test was used to compare the medians for continuous variables. Chi square test was used to determine the association between a set of independent categorical variables and IPT status in bivariate analysis.

The overall TB incidence rate was computed as the total number of TB cases per total person time recorded. Incidence rate ratio (IRR) was used to compare TB incidence rates between IPT and Non-IPT groups. Multivariable Cox proportional hazards regression model was used to obtain the factors associated with incident TB after accounting for the baseline differences of the two groups. We constructed the model by adding one variable at a time. The decision to keep a variable in the model was done if *P*-value was 0.05 and removal from the model was decided at P-value of more or equal to 0.15. Finally, Kaplan-Meiyer survival curves were used to estimate cumulative probabilities of being TB free across explanatory variables and then tested using log-rank test. To adjust for the clustering effect of the multi-site level correlation of data, each health facility was considered as a primary sampling unit and proper adjustments were considered during data analysis.

## Results

### Characteristics of study participants

The demographic and clinical characteristics of the study participants are shown in Table 1. A total of 68,378 HIV positive adults were studied. Majority 51,486 (75.3%) were females. Most of the participants 41,090 (60.1%) were from Kinondoni district. The median age at enrolment was 35.0 (Inter-quartile range, IQR = 29.3–41.8) years. Majority 26,648 (39%) were aged between 26 and 35 years. The mean body mass index (BMI) was 23.3 (standard deviation, SD = 5.2) kg/m^2^. Majority 27,704 (53.2%) of the patients had normal BMI. The median CD4+ count was 212 (IQR = 118–491) cells/μl. A large proportion (39%) of the patients had CD4+ count above 350 cells/μl. During the study period 6454 (9.4%) patients received Isoniazid preventive therapy (IPT). We compared baseline characteristics between patients who ever received IPT and those who never received IPT during the study (Table 1). We found that, there was a statistically significant difference in all of the characteristics between the two groups except for sex (*P* = 0.086) mean BMI (*P* = 0.534) and median CD4 cell count (*P* = 0.064).

**Table 1:** Socio demographic and clinical characteristics of the study participants (*N* = 68,378)

	Total	Ever received IPT (*n* = 6454)	Never received IPT (*n* = 61,924)	χ^2^-*P*-value*
Characteristic	n (%)	n (%)		
Median Age in years (IQR)	35.0 (29.3–41.8)	36.8 (31.5–45.5)	34.7 (29–41.6)	0.001
Age category (years)				0.001
15–25	7500 (11.0)	362 (5.6)	7138 (11.5)	
26–35	26,648 (39.0)	2250 (34.9)	24,398 (39.4)	
36–45	22,208 (32.5)	2468 (38.2)	19,740 (31.9)	
45+	12,022 (17.6)	1374 (21.3)	10,648 (17.2)	
Sex				0.086
Males	16,892 (24.7)	1651 (25.6)	15,241 (24.6)	
Females	51,486 (75.3)	4803 (74.4)	46,683 (75.4)	
Marital status				0.001
Single	19,249 (28.1)	1749 (27.1)	17,500 (28.3)	
Married/co-habiting	33,224 (48.6)	3094 (47.9)	30,130 (48.7)	
Widowed/divorced	8823 (12.9)	991 (15.4)	7832 (12.6)	
BMI categories (Kg/m^2^)				0.001
Underweight (< 18.5)	8054 (11.8)	815 (12.6)	7239 (11.7)	
Normal (18.5–24.5)	27,704 (40.5)	2984 (46.2)	24,720 (39.9)	
Overweight (25–29.9)	10,923 (16.0)	1136 (17.6)	9787 (15.8)	
Obese (≥30)	5446 (8.0)	547 (8.5)	4899 (7.9)	
Mean BMI (SD)	23.3 (5.2)	23.2 (5.0)	23.2 (5.2)	0.534
CD4+ categories (cells/μl)				0.001
< 200	12,891 (18.9)	1438 (22.3)	11,453 (18.5)	
200–350	9918 (14.5)	1317 (20.4)	8601 (13.9)	
> 350	14,585 (21.3)	1590 (24.6)	12,995 (21.0)	
Median CD4+ cell count (IQR)	252 (118–491)	245 (119–390)	253 (119–417)	0.064
WHO staging for HIV/AIDS				0.001
I	22,530 (32.9)	1411 (21.9)	20,664 (36.1)	
II	13,881 (20.3)	1587 (24.6)	11,652 (20.3)	
III	25,894 (37.9)	2952 (45.7)	19,957 (34.9)	
IV	4485 (6.6)	308 (4.8)	3666 (6.4)	
Cotrimoxazole use				0.001
Yes	63,041 (92.2)	6389 (99.0)	56,652 (91.5)	
No	5337 (7.8)	65 (1.0)	5272 (8.5)	
ART use				0.001
Yes	56,245 (82.3)	6214 (96.3)	50,031 (80.8)	
No	12,133 (17.7)	240 (3.7)	11,893 (19.2)	
Functional status at enrolment				0.001
Ambulatory	1449 (2.1)	58 (0.9)	1391 (2.2)	
Bedridden	381 (0.6)	35 (0.5)	346 (0.6)	
Working	66,070 (96.6)	6324 (98.0)	59,746 (96.5)	
Health facility type				0.001
Dispensary	25,858 (37.8)	165 (2.6)	25,693 (41.5)	
Health center	18,961 (27.7)	1836 (28.4)	17,125 (27.7)	
Hospital	21,407 (31.3)	4435 (68.7)	16,972 (27.4)	
Health facility ownership				
Government (Public)	60,596 (88.6)	6338 (98.2)	54,258 (87.6)	0.001
Private/FBO	6590 (9.7)	108 (1.7)	6482 (10.5)	

Some variables had missing records *Chi-squared *P*-value

### TB incidence rates

A total of 3124 TB cases were recorded during the study. The median follow-up time between patients on IPT and Non-IPT was (1.3 (IQR = 0.3–1.3) and 3.4 (IQR = 1.9–3.8) respectively. In total, 6454 patients who received IPT and 61,924 patients who did not received IPT contributed to 114,926 person-years of follow-up. The overall TB incidence rate was 2.7/100 person-years (95% CI; 2.6–2.8) (Table 2). The incidence rate of TB was 48% lower amongst patients who received IPT as compared to those who never received IPT (IRR 0.52, 95%; 0.46–0.59) (Table 2).

**Table 2:** Comparison of TB incidence rates among patients ever on IPT against those never on IPT (*N* = 62,983)

IPT status	Number of TB cases	Total person-years (TPYrs)	Incidence rate (per 100pyrs)	IRR (95% CI)
Never on IPT	2845	96,845	2.94	1.0
Ever on IPT	279	18,081	1.54	0.52 (0.46–0.59)
Total	3124	114,926	2.7	

**Fig. 2:**
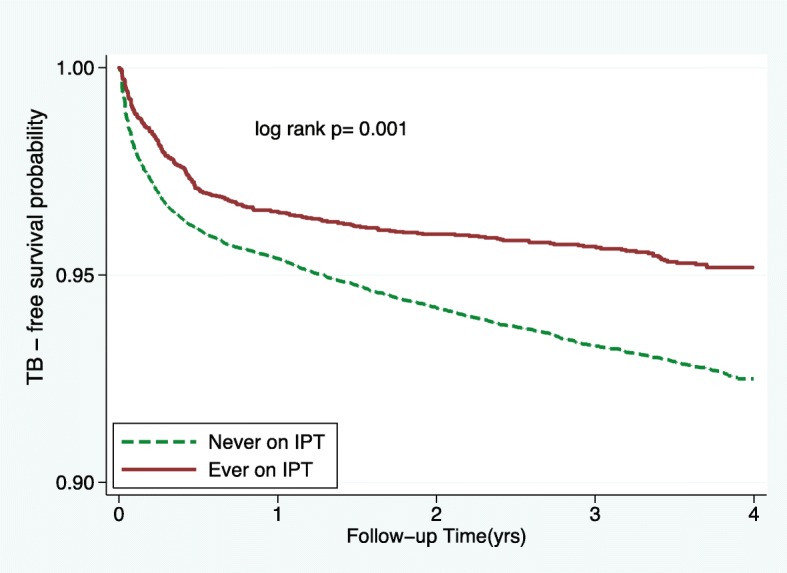
Kaplan-Meir probability of TB-free survival by IPT status

A comparison for the probability of being TB free at the end of follow-up period between IPT and Non-IPT patients is shown in Fig. 2. Patients who received IPT had higher probability of being TB free at the end of follow up period compared to those who never received IPT (log rank *P* = 0.001).

### Cox proportional hazards modelling

In the bivariable analysis, male sex, WHO stage, ART use, Cotrimoxazole use, BMI and Functional status at baseline were significantly associated with increased hazards of developing TB while IPT use, baseline CD4 count and marital status offered protection against TB acquisition (Table 3). In multivariate analysis, we adjusted for sex, marital status, baseline CD4 count, WHO stage and functional status at baseline. A number of factors remained significant predictors for incident TB. Factors associated with significantly increased risk for TB included male patients (aHR = 1.8, 95% CI; 1.6–2.0) compared to females, WHO stage III (aHR = 2.7, 95% CI; 2.3–3.3 and IV (aHR = 2.4, 95% CI; 1.9–3.1) compared to patients with WHO stage I, being underweight (aHR = 1.7, 95% CI; 1.5–1.9) compared to normal weights as well as history of using ART (aHR = 1.5, 95% CI; 1.0–2.2) and cotrimoxazole (aHR = 2.2, 95% CI; 1.4–3.5) compared to those never used ART or cotrimoxazole respectively. On the other hand, overweight (aHR = 0.7, 95% CI; 0.6–0.8), obese patients (aHR = 0.5, 95% CI; 0.4–0.7), patients with baseline CD4 count between 200 and 350 cells/μl (aHR = 0.7, 95% CI; 0.6–0.8) and above 350 cells/μl (aHR = 0.5, 95% CI; 0.4–0.6) as well as patients ever on IPT (aHR = 0.6, 95% CI; 0.5–0.8) were associated with significantly lower risk of developing TB. On assessing the effect of IPT on TB incidence for patients ever on ART and those not on ART, we found that among patients ever on ART, IPT was associated with 40% (aHR = 0.6, 95% CI; 0.5–0.7) reduced risk of developing TB as summarized in Table 4 below.

**Table 3:** Cox proportional hazards analysis of factors associated with incident tuberculosis (N = 62,983)

Characteristic	Unadjusted		Adjusted^¶^	
	HR	95% CI	aHR	95% CI
Age (years)				
15–25	1.0		1.0	
26–35	1.6	1.3–1.9	1.6	1.0–2.4
36–45	2.1	1.8–2.5	1.6	1.2–2.2
45+	2.2	1.7–2.6	1.3	0.9–2.0
Sex				
Females	1.0		1.0	
Males	2.4	2.2–2.6	1.8	1.7–1.9
Marital status				
Single	1.0		1.0	
Married/co-habiting	0.8	0.7–0.9	0.9	0.8–1.0
Widowed/divorced	1.1	1.0–1.2	0.9	0.8–1.1
BMI categories (Kg/m^2^)				
Underweight (< 18.5)	2.5	2.3–2.7	1.8	1.5–2.1
Normal (18.5–24.5)	1.0		1.0	
Overweight (25–29.9)	0.5	0.4–0.6	0.7	0.6–0.8
Obese (≥30)	0.4	0.3–0.4	0.5	0.4–0.7
CD4 categories (cells/μl)				
< 200	1.0		1.0	
200–350	0.5	0.5–0.6	0.7	0.6–0.8
> 350	0.3	0.3–0.4	0.5	0.4–0.6
WHO clinical staging of HIV				
I	1.0		1.0	
II	2.0	1.7–2.3	1.4	1.2–1.6
III	4.4	3.9–5.0	2.7	2.2–3.2
IV	5.3	4.5–6.1	2.6	1.8–3.9
Cotrimoxazole use				
No	1.0		1.0	
Yes	2.4	1.9–3.1	2.2	1.3–3.9
IPT status				
Ever	0.7	0.6–0.7	0.6	0.4–0.9
Never	1.0			
ART status				
Ever	2.6	2.1–3.1	1.5	1.0–2.2
Never	1.0		1.00	
Functional status at enrolment				
Working	1.0		1.0	
Bedridden	1.8	1.2–2.8	2.5	2.0–3.2
Ambulatory	6.1	5.3–7.0	1.2	0.4–3.2

adjusted for the effect of age, sex, BMI, CD4 count, WHO stage, IPT use, ART status and Functional status at enrollment

**Table 4:** The effect of IPT on TB incidence rate among patients by ART status

	Characteristic	Hazards ratio (95% CI)	*P*-value
Ever on art (*N* = 56,245)	Ever on IPT	0.6 (0.5–0.7)	0.001
	Never on IPT	Ref	
Never on art (*N* = 12,133)	Ever on IPT	0.5 (0.2–1.7)	0.298
	Never on IPT	Ref	

## Discussion

The overall TB incidence rate of 2.7 per 100 person years in our study is about three fold lower than previous reported by a study in Dar es salaam region [13] . The IPT uptake in our study was only 9.4%. However, TB incidence rate was 48% lower among patients on IPT compared with non-IPT patients. Being male, underweight (BMI < 18.5 kg/m2), higher WHO HIV clinical staging (II-IV), use of cotrimoxazole and ART were significantly associated with increased incident TB in the studied population.

The overall TB incidence rate found in this study is similar to what has been reported by other studies in Sub Saharan Africa which used routine HIV program data [14–16]. The use of HIV program data makes the findings more reliable as the data is collected in real-time and in most cases it is large enough to offer sufficient power to the study. Furthermore, the observed TB incidence rate might imply that most countries in Sub Saharan Africa are experiencing a relatively stable TB incidence given the wide scale-out of various TB preventing interventions including IPT.

The low level of IPT uptake in our study is similar to what has been documented for other countries in Sub Saharan Africa [17]. This may be due to regular stock outs and other challenges involved in the supply chain of Isoniazid during the roll out. However, we did not explore the reasons for low IPT uptake in our study.

In the present study, we found that IPT reduces the TB incidence rate among HIV infected adults by almost 50%. Even after adjusting for potential confounders in the multivariable analysis we still found that IPT had a protective effect on TB incidence. The effectiveness of IPT is consistent to findings in Brazil [7] and Ethiopia [16, 18]. We further noted that patients on IPT had a higher TB free survival time compared to non-IPT patients. This finding is in agreement to that reported in South Africa and Ivory Coast [19, 20]. Despite our study being an observational in nature, but still it reconfirms and adds to the body of knowledge on the effectiveness of IPT in reducing TB burden among HIV infected individuals.

Risk factors for incident TB in our this study are consistent with those reported in previous studies [13, 21–24]. We found that being male, BMI, baseline CD4 count, advanced WHO stage, ART use and Cotrimoxazole use were significantly associated with an increased risk of TB incidence.

There are still no clear reasons as to why being male is associated with higher risk of developing TB. One possible explanation from our study could be the fact that most females were enrolled through PMTCT option B+ thus had a protective effect of prior ART exposure and majority of males were enrolled with advanced disease stage. In contrast with previous studies [4, 13] the history of using ART alone during follow up did not offer a protective effect against incident TB. However, our study found that the protective effect of IPT against TB was significantly marked among patients ever received ART and this is similar to what have been documented in other studies [19, 24].

### Strengths and limitations of the study

This is one of the few studies in Tanzania which demonstrated the effect of IPT on TB incidence using routine HIV program data. The strength of this study relies on the fact that it involved a large cohort of patients from different facilities (multi-sites). However, our study had some limitations which need to be considered while interpreting the results. Firstly, the eligibility criteria for IPT initiation undertaken in this analysis did not consider past exposure to Isoniazid or repeated course of IPT (i.e. after two years as recommended). This might underestimate the IPT uptake in this study. Secondly, the shorter follow-up time for patients on IPT limited the observed TB cases in this group; possibly we could have observed many cases given a longer follow up time. Thirdly, we did not also assess the differences in loss to follow-p between those who have received IPT and those who have not. This may lead to underestimation or overestimation of the observed effect of the IPT if there is a difference in loss to follow-up between the two groups. Fourthly, we excluded patient with missing records on age status, but there was only small percentage with missing records. However, due to the small percentage of missing records on age, the effect of this exclusion on our finding is unlikely. Fifth, information on routine drug use such as IPT, ART and cotrimoxazole used in this study did not consider details on completion, defaulting or adherence levels during the drug course. Such missing information limits the comparability and generalizability of our findings to the larger population.

## Conclusion and recommendations

IPT has been shown to decrease the TB incidence rate compared to non-IPT patients regardless of ART use. This suggests the need for IPT to be made available and continuously be given to HIV infected individuals after ruling out active TB. Some factors such as low BMI, advanced WHO stage were associated with an increased risk of TB among HIV infected individuals. This implies that in-depth TB screening and regular follow up should be done among these patients. Future studies should account for factors such as drug adherence and completion status to capture the true effect of IPT on TB incidence as well as involve more geographical regions to represent the whole country.

## Abbreviations

AHR: Adjusted hazards ratio
ART: Antiretroviral therapy
CI: confidence interval
CTC: Care and treatment clinics
HIV: Human Immunodeficiency virus
HR: Hazards ratio
IPT: Isoniazid preventive therapy
IQR: Inter quartile range
KCMUCO: Kilimanjaro Christian Medical University College
MOHCDGEC: Ministry of Health, Community Development, Gender Elderly and Children
PMTCT: Prevention of Mother To Child Transmission of HIV/AIDS
TB: Tuberculosis
WHO: World Health Organization
